# Ein After Action Review auf kommunaler Ebene: Lessons learned in der Corona-Einheit des Gesundheitsamtes Düsseldorf infolge der Covid-19-Pandemie, März 2023

**DOI:** 10.1055/a-2329-7058

**Published:** 2024-10-24

**Authors:** Hannah Höglund-Braun, Anne Lisa Quartey, Maya Ganter, Lutz Ehlkes, Steffen Geis, Max Skorning, Sybille Rehmet, Anna Kuehne

**Affiliations:** 1 Stabsstelle Gesundheitlicher Bevölkerungsschutz, Landeshauptstadt Düsseldorf Gesundheitsamt, Düsseldorf, Germany; 2 Postgraduiertenausbildung für Angewandte Epidemiologie (PAE), Robert-Koch Institut, Berlin, Germany; 3 European Programme for Intervention Epidemiology Training (EPIET), ECDC, Stockholm, Sweden; 4 Abteilung Gesundheitsschutz und Gesundheitsaufsicht, Landeshauptstadt Düsseldorf Gesundheitsamt, Düsseldorf, Germany; 5 Stabsstelle Krankenhaushygiene, Universitätsklinikum Düsseldorf, Düsseldorf, Germany; 6 Amtsleitung, Landeshauptstadt Düsseldorf Gesundheitsamt, Düsseldorf, Germany; 7 Lehrstuhl Öffentliche Gesundheit, Zentrum für Evidenzbasierte Gesundheitsversorgung (ZEGV), Universitätsklinikum und Medizinische Fakultät Carl Gustav Carus der TU Dresden, Desden, Germany

**Keywords:** After-Action Review, COVID-19, Pandemie, Public Health, Krisenmanagement, Pandemie-Vorbereitung, After-Action Review, COVID-19, Public Health, pandemic preparedness, crisis management, Germany

## Abstract

**Hintergrund** After-Action Reviews (AARs) stellen strukturierte, qualitative Evaluationen von Krisenmaßnahmen dar. Wir beschreiben hier die Durchführung eines AARs zur Evaluation der Corona-Einheit des Gesundheitsamtes Düsseldorf.

**Methoden** Wir nutzten für das AAR das Format der Arbeitsgruppe, das durch semi-strukturierte Interviews mit Schlüsselpersonen ergänzt wurde. Dabei orientierten wir uns an den Leitlinien für AARs der Weltgesundheitsorganisation (WHO) und des Europäischen Zentrums für die Prävention und die Kontrolle von Krankheiten (ECDC).

**Ergebnisse** Die Teilnehmenden identifizierten neun relevante Herausforderungen mit Verbesserungspotential, die drei Kategorien zugeordnet werden konnten: (I) Herausforderungen in der Bereitstellung angeforderten Personals, (II) übergeordnete Koordinationsfragen und (III) häufige Umzüge. Die Teilnehmenden entwickelten folgende Lösungsvorschläge für diese Herausforderungen: (I) 1. regelmäßiger ämterübergreifende Abfrage von freiwillig einsatzbereitem Personal, 2. Berücksichtigung von abrufbaren Ressourcen städtischer Tochterunternehmen und Einbindung von Personal mit Leitungserfahrung anderer Ämter, 3. Koordination von Bereitstellung und Onboarding, 4. Vorschlag zu früher Kommunikation in Bezug auf unterschiedliche Vergütungsregelungen, (II) 5. Etablierung einer übergeordneten Koordinationsinstanz für die Kriseneinheit, 6. klarere Zuständigkeitsabgrenzung innerhalb der Kriseneinheit mit eindeutigen Aufgabenprofilen, 7. Etablierung einer gemeinsamen Arbeitsoberfläche sowie die 8. Etablierung eines zentralen Dashboards zur Generierung aller Lageberichte und (III) 9. Identifizierung eines Gebäudes zur Unterbringung einer Kriseneinheit im Krisenfall.

**Schlussfolgerung** Diese Studie stellt eine der ersten Anwendungen eines AARs zur Evaluation von Krisenstrukturen im Öffentlichen Gesundheitsdienst (ÖGD) auf kommunaler Ebene im Kontext der COVID-19-Pandemie dar. Während einige der identifizierten Herausforderungen Düsseldorf-spezifisch sind, waren viele Kommunen während der COVID-19-Pandemie mit Herausforderungen in den Bereichen der Personalgenerierung und Koordination konfrontiert; die entwickelten Lösungsansätze können entsprechend auch jenseits von Düsseldorf von Relevanz sein. Kommunale Krisenpläne können in Bezug auf die identifizierten Herausforderungen geprüft und entsprechend angepasst werden. Der Austausch von Evaluations-ergebnissen unter den ÖGD-Akteuren kann dazu beitragen, die Krisenstrukturen langfristig zu stärken.

## Abstract (en)

**Background** After-Action Reviews (AARs) represent structured, qualitative evaluations of crisis interventions. Here, we describe the implementation of an AAR to assess the Corona Unit of the Duesseldorf Health Department.

**Methods** We employed an after-action review (AAR) using the working group format supplemented by structured interviews with key personnel. Our approach was guided by the guidelines for AARs provided by the World Health Organization (WHO) and the European Centre for Disease Prevention and Control (ECDC).

**Results** Participants identified nine relevant challenges with potential for improvement in three categories: (I) challenges in recruiting requested personnel, (II) overarching coordination issues, and (III) frequent relocations. Participants proposed solutions for these challenges: (I) 1. Routine cross-agency inquiries for voluntarily available personnel, 2. consideration of resources from municipal subsidiaries and involvement of personnel with leadership experience from other departments, 3. Better coordination of deployment and onboarding, 4. proposal for early communication regarding different compensation regulations, (II) 5. establishment of an overarching coordination authority for the crisis unit, 6. clearer delineation of responsibilities within the crisis unit with explicit job profiles, 7. establishment of a shared working platform, and 8. creation of a central dashboard for generating all situation reports. Additionally, they identified (III) 9. a facility for housing a crisis unit in case of an emergency.

**Conclusion** This study represents one of the first applications of an AAR for evaluating crisis structures at the level of a local Public Health Authority (LPHA) in the context of the COVID-19 pandemic. While some of the identified challenges are specific to Duesseldorf, many municipalities faced personnel recruitment and coordination challenges during the COVID-19 pandemic. Municipal crisis plans can be reviewed and adjusted concerning the identified challenges. Sharing evaluation results among LPHA stakeholders can contribute to strengthening crisis structures in the long term.

## Einleitung

Die COVID-19-Pandemie (2020–2023) stellt ein in ihrem Ausmaß im 21. Jahrhundert unvergleichliches Ereignis dar. Nahezu jeder Lebensbereich wurde durch die Auswirkungen dieser globalen Krise berührt. Gesundheits-, Mobilitäts- und Wirtschaftssysteme wurden weltweit einer bis dato nicht gekannten Belastung ausgesetzt 1 .

Die Meldung hunderter und zeitweise tausender Neuinfektionen pro Tag in den deutschen Stadt- und Landkreisen 2 sowie die Vorgaben aus regelmäßig an die aktuelle Lage angepassten Verordnungen durch Land und Bund, erforderte zur Bewältigung der Lage kommunale Strukturen, die im erforderlichen Ausmaß noch nicht existierten 3 . Vor diesem Hintergrund war das Gesundheitsamt Düsseldorf bestrebt, ein belastbares System zum Corona-Management zu etablieren, welches bei schwankenden und rasch steigenden Fallzahlen skalierbar und gleichzeitig so belastbar war, dass auch ein zuverlässiger Langzeit-Einsatz gewährleistet werden konnte. So entstand in Düsseldorf die Einheit des „Corona-Case-Management (CCM)“. In verschiedenen Untereinheiten wurden infektionsepidemiologische Surveillancedaten erfasst, Indexfälle registriert und Kontaktpersonen ermittelt, Lageberichte verfasst, vulnerable Gruppen durch spezialisierte Teams betreut (bspw. Kindertagesstätten, Seniorenpflegeeinrichtungen) sowie Presse- und Bürgeranfragen beantwortet und der enge Austausch mit dem Krisenstab der Stadt gehalten. In enger Kooperation mit der städtischen Berufsfeuerwehr wurde durch diese ein großes Test- und ein Impfzentrum aufgebaut.

Nachdem im März 2023 alle vorherigen Verordnungen aufgehoben wurden, führten wir ein After-Action Review (AAR) 4 5 6 7 8 9 10 11 durch, um die Effektivität dieser Einheit zu evaluieren.

### Zielsetzung des After-Action Reviews

Ziel unseres AARs ist es, bewährte Prozesse und Stärken der CCM-Struktur aufzuzeigen, sowie Lücken, Herausforderungen und Lösungsansätze zu identifizieren und im Einklang mit dem Krisenmanagementzyklus zu evaluieren 11 12 [Bibr RGESU-2023-11-1966-OA-0013] [Bibr RGESU-2023-11-1966-OA-0015] 16 17 .

## Methodik

Ein After-Action Review (AAR) ist eine strukturierte, qualitative Überprüfung der Maßnahmen, die als Reaktion auf ein bestimmtes Krisenereignis durchgeführt werden 4 5 6 7 8 9 10 und ein Teil des „Monitoring and Evaluation Frameworks“ der Weltgesundheitsorganisation (WHO) 5 9 11 .

**Abb. 1:**
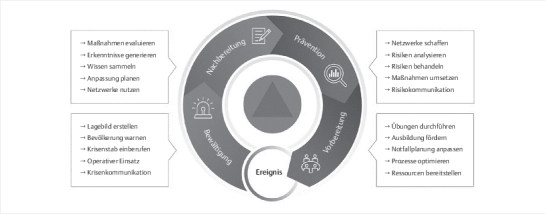
Krisenmanagement-Zyklus 11 12 13 (Quelle: Bundesamt für Bevölkerungsschutz und Katastrophenhilfe [BBK]).

Dabei dient der AAR als Mittel zur Identifizierung und Dokumentation bewährter Praktiken und Herausforderungen eines Prozesses der Krisenbewältigung und stellt einen essentiellen Schritt im Krisenmanagements-Zyklus dar ( Abb. 1 ).

Der Mehrwert eines AARs ergibt sich aus der Betonung des kollektiven Lernens und des Erfahrungsaustauschs, wobei das Wissen der beteiligten Personen im Fokus steht 5 7 9 . Die WHO schlägt vier Methoden für die Durchführung eines AAR vor: (i) Debriefings/Nachbesprechungen, (ii) Arbeitsgruppen, (iii) Interviews mit Schlüsselinformanten und (iv) Studien mit gemischten Methoden 5 7 9 . Die Endergebnisse sollten in einem qualitativen Format zusammengefasst werden 5 7 9 .

Die Überprüfungen der Prozesse ist nicht dazu gedacht, die Leistung oder Kompetenz des Einzelnen zu bewerten oder Schuldzuweisungen zu machen, sondern Lernmöglichkeiten aufzeigen und zum Zyklus der kontinuierlichen Qualitätsverbesserung der Notfallvorsorge und -planung beizutragen 5 7 9 .

Es wird empfohlen, einen AAR 3–6 Monate nach Ende eines entsprechenden Ereignisses durchzuführen, um sicherzustellen, dass die wichtigsten Akteure noch zugänglich sind und das Ereignis noch in guter Erinnerung haben 5 7 .

Für die Konzeption, Vorbereitung, Durchführung und Nachbereitung des AAR wurden die von WHO und ECDC bereitgestellten generischen Vorlagen als Basis genutzt und angepasst 5 7 .

### Umsetzung des AARs in Düsseldorf

Ende März 2023 führten wir ein AAR in einer vierstündigen Präsenzveranstaltung im Gesundheitsamt Düsseldorf im Workshop-Format durch, welches durch semi-strukturierte Interviews mit Schlüsselpersonen ergänzt wurde.

Das Organisationsteam bestand aus drei Personen, die im Verlauf des Workshops eine moderierende, dokumentierende und neutrale Position einnahmen. Durch ihre Einbindung in den CCM-Einheiten während des gesamten Pandemieverlaufs fiel die in den offiziellen Dokumenten 5 7 angegebene lange Vorbereitungszeit (bis zu 6 Monate) erheblich kürzer aus.

Im Vorfeld der Durchführung wurden die relevanten Leitungskräfte identifiziert.

Aufgrund der postpandemischen Einbindung aller Schlüsselpersonen in die Regeltätigkeiten war die Durchführung des AARs nur in Form eines vierstündigen Workshops möglich. Die von ECDC und WHO bereitgestellten Vorlagen und Empfehlungen 5 7 wurden an dieses zeitliche Format angepasst, Triggerfragen entsprechend vorbereitet und der Rahmen auf die Betrachtung der internen Herausforderungen klar formuliert. Der Workshop gliederte sich in drei Teile.

Im ersten Teil wurden Beispiele guter Praxis besprochen, um eine positive Arbeitsatmosphäre zu schaffen und die Teilnehmer dazu zu ermutigen, die Stärken der neuen Strukturen zu erkennen, die später Teil der Lösungsansätze sein könnten.

Im zweiten Teil wurden in Kleingruppen Herausforderungen, nicht erzielte Maßnahmenwirkungen und Vorbereitungslücken erarbeitet. Die Teilnehmenden wurden gebeten sich aufgrund der limitierten Zeit auf die internen Herausforderungen zu konzentrieren.

Nach Abschluss des Workshops wurden die erarbeiteten Ergebnisse in einem Abschluss-Protokoll zusammengefasst und den Teilnehmenden zur Validierung vorgelegt.

## Ergebnisse

Insgesamt nahmen 10 Personen am Workshop teil. Hierbei handelte es sich um die Gesamtleitung des CCM sowie die Leitungskräfte der verschiedenen Untereinheiten des CCMs (es handelt sich hierbei um Ärzt*innen, Epidemiolog*innen, Hygienekontrolleur*innen, Verwaltungsfachwirt*innen, Leitungskräfte der städtischen Feuerwehr, Quereinsteiger*innen BWL mit Führungserfahrungen und IT-Fachkräfte).

Drei Leitungskräfte, die den Termin nicht in Präsenz wahrnehmen konnten, wurden im Anschluss in 30–60-minütigen Telefoninterviews anhand der Struktur des Workshops befragt und die Antworten wurden in das Protokoll integriert.

Die Teilnehmenden identifizierten neun relevante Herausforderungen mit Verbesserungspotential, die drei Kategorien zugeordnet werden konnten: Herausforderungen in der Bereitstellung angeforderten Personals, übergeordnete Koordinationsinstanz und häufige Umzüge. Die Teilnehmenden konnten im Rahmen des AARs zu allen Ausgangslagen und Herausforderungen Verbesserungspotentiale identifizieren und Lösungsvorschläge erarbeiten (s. Tab. 1 ).

**Table:** **Tab. 1** Zusammenfassung der 3 Kategorien mit 9 Herausforderungen die von Teilnehmenden des AARs als konkrete Verbesserungspotentiale identifiziert wurden.

Ausgangslage	Identifizierte Verbesserungsmöglichkeiten
***I. Ausgangslage und Herausforderungen in der Bereitstellung angeforderten Personals***	
1. *Langsame Personalbereitstellung*	Regelmäßige ämterübergreifende Abfrage der Einsatzbereitschaft von Mitarbeiter*innen im Krisenfall
2. *Eignung entsendeten Personals*	Wertvolle Ressourcen der Mitarbeiter*innen städtischer Tochterunternehmen (Personal- und Materialressourcen) mit bedenken.
3. *Onboarding angeforderten Personals*	Koordiniertes Onboarding (Personalamt→Kriseneinheit) sichert effizienten Personaleinsatz.
4. *Uneinheitliche Vergütungsregelungen*	Frühe Kommunikation zu unterschiedlichen Vergütungsregelungen.
***II. Übergeordnete Koordinationsinstanz***	
5. *Fehlen einer übergeordneten Koordinationsinstanz*	Etablierung einer übergeordneten Koordinationsinstanz.
6. *Unklare Zuständigkeitsbereiche unter den Einheiten*	Klarere Abgrenzung von Zuständigkeitsbereichen mit Hinterlegung eindeutiger Aufgabenprofilen.
7. *Fehlen einer gemeinsamen Arbeitsoberfläche*	Etablierung einer eigenen digitalen Arbeitsoberfläche.
8. *Viele Lageberichte aus den einzelnen Einheiten*	Etablierung eines zentralen Dashboards zur Generierung aller Lageberichte.
***III. Häufige Umzüge***	
9. *Fehlendes Gebäude für eine Kriseneinheit*	Identifizierung eines Gebäudes mit Potenzial zur Unterbringung einer großen Kriseneinheit im Krisenfall.

I. Ausgangslage und Herausforderungen bei der Bereitstellung angeforderten Personals:1.

1. **Langsame Bereitstellung angeforderten Personals** Teilweise dauerte es mehrere Wochen, bis in den verschiedenen Ämtern Personal identifiziert wurde, welches entsendet werden konnte. Auch variierte die Bereitschaft zur langanhaltenden Unterstützung. Identifizierte Verbesserungspotentiale: Vorschlag zur Durchführung regelmäßiger ämterübergreifende Abfragen, welche Mitarbeiter*innen im Krisenfall freiwillig einsatzbereit sind, um eine schnellere und, falls erforderlich, langfristige Bereitstellung von Personal zu erreichen.
2. **Eignung entsendeten Personals für die neuen Aufgaben** Die Teilnehmenden berichteten, dass der telefonische Kontakt zu Bürger*innen für einige der entsendeten Personen trotz vorbereitender Schulungen eine Herausforderung darstellte. Das Personal städtischer Tochterunternehmen (bspw. aus dem Veranstaltungsmanagement) hingegen, deren Arbeitsfähigkeit durch bestehende Verordnungen zeitweise stark eingeschränkt bis unmöglich war und welches ebenfalls entsandt wurde, verfügte häufig über viel Erfahrung im Kontakt mit Menschen, planerische Fähigkeiten und materielle Ressourcen zur Lenkung großer Menschenmengen aus Eventmanagement und Messebau. Diese Fähigkeiten wurden in der Kontaktpersonennachverfolgung und Planung großer Test- und Impfzentren als sehr unterstützend und wertvoll wahrgenommen. Auffällig, wenn auch vor dem Hintergrund des Erhalts der eigenen Funktionsfähigkeit verständlich, war, dass wenig Leitungspersonal anderer Ämter bereitgestellt wurde. Da auch die Kriseneinheit hierarchisch strukturiert ist, hat sich aus Sicht der Teilnehmenden die Einbindung von Leitungspersonal anderer Ämter zur Besetzung von Positionen mit entsprechendem Anforderungsprofil als sinnvoll und effizient erwiesen. Identifiziertes Verbesserungspotential: Bei Bereitschaftsabfragen sollten die Ressourcen der Mitarbeiter*innen städtischer Tochterunternehmen (Personal und Material) mit bedacht werden. Eine Bereitschaftsabfrage kann mit der Bitte, auch Personal mit Leitungserfahrung zu berücksichtigen, verknüpft werden.
3. **Koordiniertes Onboarding des angeforderten Personals** Die Teilnehmenden erläuterten, dass es neben Phasen, langsamer Personalbereitstellung auch Phasen gab, in denen in sehr kurzer Zeit 60–70 Personen aufgenommen werden musste. Neben Problemen in Bezug auf die technische Ausstattung (Laptops, Headsets), bestanden Schwierigkeiten so große Gruppen neuer Kolleg*innen adäquat einzuarbeiten (z. B. SurvNet-Schulungen). Dies erschwerte das adäquate Onboarding, beeinflusste die Qualität der Aufgabenerfüllung negativ und führte zu Unzufriedenheit sowohl beim neuen Personal, als auch bei den Personen, die das Onboarding durchführten. Identifiziertes Verbesserungspotential: Die Teilnehmenden wünschten sich eine bessere Koordination und engere Absprachen zwischen Personalbereitstellung und den vorhandenen Kapazitäten zum Onboarding, um einen möglichst schnellen und effizienten Einsatz neuer Mitarbeitenden sicherzustellen.
4. **Uneinheitliche Vergütungsregelungen** Die Teilnehmenden aller Untereinheiten nannten die teilweise uneinheitliche Vergütung für gleiche Arbeit, unterschiedliche Regelungen zur Zeiterfassung, sowie Möglichkeiten/Nichtgestattung zur Wochenendarbeit als Herausforderungen in der Personalführung. So wurde beklagt, dass einige Personengruppen die besser vergütete Wochenendarbeit leisten durften, anderen war dies nicht gestattet. Identifiziertes Verbesserungspotential der Teilnehmenden: Aufgrund der verschiedenen Personengruppen (Beamte, Angestellte, Nachwuchskräfte, Studierende, etc.), die in Krisenzeiten unterstützen, sollte eine Kommunikation in Bezug auf unterschiedliche Vergütungsregelungen frühzeitig erfolgen. So können Arbeitszeitregelungen in verschiedenen Teams (Beamte, Angestellte) variieren oder die Vergütung kann unterschiedlich erfolgen, was in der Regel vom Tarifsystem abhängig ist.

**II. Übergeordnete Koordination:**

5. **Fehlen kontinuierlicher übergeordneter Koordinationsinstanz** Die Teilnehmenden nannten das Fehlen einer kontinuierlichen übergeordneten Koordinationsinstanz als Herausforderung. Es fehlte eine Funktion, die bei der stetig und teilweise rasant wachsenden Kriseneinheit Koordinierungs- und Steuerungsaufgaben zuverlässig übernahm. Eine solche Funktion war nicht dauerhaft vorhanden. Dadurch kam es mitunter zu Schnittstellenproblematiken und der Notwendigkeit eingeführte Prozesse kurzfristig erneut zu überarbeiten. Identifiziertes Verbesserungspotential der Teilnehmenden: Etablierung einer übergeordneten Koordinationsinstanz. Diese Funktion sollte den Organisationseinheiten übergeordnet sein. Die Funktion kann den Aufbau und Zuwachs einzelner Einheiten überblicken, Änderungsprozesse einleiten und deren Auswirkungen auf andere Einheiten überblicken, um Reibungsverluste zu minimieren.
6. **Unklare Zuständigkeitsbereiche unter den Einheiten** In der Diskussion mit den Teilnehmenden stellte sich heraus, dass im Pandemieverlauf die Zuständigkeiten einzelner Einheiten bei den Beteiligten nicht immer klar benannt waren. So kam es teilweise zu paralleler Aufgabenbearbeitung. Hier wurde besonders eine mangelnde Kommunikation untereinander als Problem identifiziert. Ebenso wurde in einigen Einheiten eine bessere fachliche Anbindung an das Gesundheitsamt gewünscht. Identifizierte Verbesserungspotentiale: Klarere Abgrenzung von Zuständigkeitsbereichen innerhalb der CCM-Einheiten mit eindeutigen Aufgabenprofilen (SOPs, Organigramme). Dabei können die im Verlauf der COVID-19-Pandemie entstandenen Stellenbeschreibungen und die in der Dokumentation der Struktur festgehaltenen Aufgabenbereiche und Anforderungen zur Spezifizierung und zur Abgrenzung genutzt werden.
7. **Fehlen einer gemeinsamen Arbeitsoberfläche** Die Teilnehmenden berichteten, dass sich im Rahmen der Corona-Pandemie mit dem Zufluss vieler neuer Mitarbeiter*innen aus verschiedenen Ämtern zeigte, dass eine gemeinsame Arbeitsoberfläche unerlässlich für einen ungestörten Ablauf und reibungslosen Informationsaustausch ist. Fehlende oder eingeschränkte Zugangsrechte der Mitarbeiter*innen erschwerten den Austausch sowohl zwischen als auch innerhalb der Einheiten. Identifiziertes Verbesserungspotential: Im Krisenfall ist ein uneingeschränkter Informationsaustausch zwingend notwendig, eingeschränkte Zugriffsrechte (Zugehörigkeit zu anderen Ämtern, Datenschutz, etc…) können alle Anstrengungen, die zur Gewährleistung eines effizienten Infektionsschutzes unternommen werden konterkarieren. Es sollte für alle Mitarbeiter*innen einer Kriseneinheit schnell und unkompliziert möglich sein, allgemeine Informationen des eigenen Teams und anderer Einheiten einzusehen. Eine gemeinsame Nutzeroberfläche könnte die angesprochenen Probleme lösen und erleichtern, schnell richtige Ansprechpartner für bestimmte Themen zu finden, wenn aktuell gehaltene Organigramme und SOPs der jeweiligen Einheiten hinterlegt sind. Dies kann ein gemeinsames Laufwerk oder eine gemeinsame Dokument-Management-Oberfläche sein, die dem Stand der Technik entsprechen sollte.
8. **Viele Lageberichte aus den einzelnen Einheiten** Die Teilnehmenden berichteten, dass das Erstellen und die Weitergabe von Lageberichten aus den verschiedenen Einheiten mit Falldatenabfragen unterschiedlicher Uhrzeiten gelegentlich zur Veröffentlichung voneinander abweichender Fallzahlen führten. Daraus resultierende Presse- und Bürgeranfragen zu Inzidenzen mussten aus den verschiedenen Untereinheiten beantwortet werden und führten zu einer Störung des Arbeitsflusses. Identifiziertes Verbesserungspotential der Teilnehmenden: Ein zentrales Dashboard mit aggregierten Daten und einem konsistenten Überblick über alle Bereiche wurde von allen Einheiten gewünscht. Die Teilnehmenden hoffen, dass dies zukünftig die Erstellung von Lageberichten erleichtert und vereinheitlicht. Presseanfragen könnten aus diesen Daten gespeist werden und Entscheidungsträger könnten sich jederzeit zu allen relevanten Bereichen informieren.

**III. Häufige Umzüge:**

9. **Fehlen eines großen Gebäudes für eine Kriseneinheit** In Arbeitsgruppe und Interviews wurde berichtet, dass Umzüge meist aufgrund wachsender Teamgrößen notwendig wurden, sie betrafen sowohl Einzelpersonen als auch ganze Teams. Die Umzüge, besonders in Phasen hoher Inzidenzen und hoher Arbeitsbelastung führten zu Einschränkungen der Funktionsfähigkeit operierender Einheiten und zu zusätzlich empfundenem Stress. Identifiziertes Verbesserungspotential: Identifizierung eines Gebäudes zur Unterbringung einer großen Kriseneinheit im Krisenfall. Im Falle einer Pandemie können Lösungen wie desk sharing oder home-office sinnvoll sein. Eine Reduktion vieler verschiedener Standorte wurde von allen Teilnehmenden befürwortet.

**Beispiele guter Praxis**

Neben den Herausforderungen, wurden von den Teilnehmenden auch Beispiele guter Zusammenarbeit und neue Bereicherungen genannt. Als besonders gut empfanden die Teilnehmenden, dass die gesamte Stadtverwaltung im durch die Krise erforderlichen Umfang zusammen an der Bewältigung der Corona-Pandemie gearbeitet hat. Als Begründung wurde die Betroffenheit und Gefährdung Aller durch das initial nicht gut bekannte neue Coronavirus genannt, sowie die sehr hohe Motivation Aller sich an der Bewältigung der Pandemiefolgen zu beteiligen. Als weitere positive Punkte wurde die enge Vernetzung verschiedener Ämter und Strukturen sowie die neu entstehenden kurzen Dienstwege zur schnellen und effizienten Problemlösung genannt. Die Diversität der neu entstehenden Teams sowie die Teamfindung als solche wurden ebenfalls sehr positiv bewertet.Es wurde positiv bewertet, dass ein schneller Personalauf- und abbau grundsätzlich möglich war, sowie, dass Engagement von Mitarbeiter*innen gesehen und belohnt wurde. Auch die engere Verknüpfung mit der Heinrich-Heine-Universität sowie der Universitätsklinik Düsseldorf durch Studien und Begleitung von Dissertationen wurde positiv bewertet. Ebenfalls als sehr positiv wurde die durch die Pandemie bedingte schnellere Digitalisierung einiger Prozesse, sowie die Einführung von SurvNet für die Kontaktpersonennachverfolgung und Demis für die Befundeingänge empfunden.

## Diskussion

Das After-Action Review des CCM in Düsseldorf stellt eine der ersten Anwendungen eines AAR zur Evaluation von Krisenstrukturen im Öffentlichen Gesundheitsdienst (ÖGD) auf kommunaler Ebene im Kontext der COVID-19-Pandemie dar.

Die Evaluation ermöglichte es Erfahrungen und Lehren auszutauschen und Herausforderungen sowie Verbesserungspotential der geschaffenen Strukturen zu identifizieren und diese gemeinsam einzuordnen. Die Möglichkeit, an einem solchen Austausch teilzunehmen und eigene Erfahrungen und Erlebnisse sowie Ideen zur Lösung von Problemen benennen zu können wurde von allen Teilnehmenden als sehr positiv wahrgenommen. Auch der zeitliche Rahmen von vier Stunden wurde vor dem Hintergrund fortbestehender Regeltätigkeiten als angemessen bewertet.

Während einige Herausforderungen spezifisch die Situation in Düsseldorf charakterisieren, sind viele beschriebenen Problemstellungen, wie Personalknappheit, die zeitige Bereitstellung angeforderten Personals, die Eignung des entsendeten Personals, transparente Vergütungsregelungen sowie eine gemeinsam zu nutzenden Arbeitsoberfläche auch für andere Gesundheitsämter beschrieben worden 18 19 20 , sowie persönliche Kommunikation mit Kolleg*innen anderer Gesundheitsämter). Die in Düsseldorf gemeinsam im Rahmen des AARs entwickelten Lösungsvorschläge können deshalb auch für kommunale Gesundheitsbehörden, die keine Möglichkeit zur Durchführung eines AARs haben, von Relevanz sein und entsprechend der jeweiligen Situation vor Ort für die Weiterentwicklung der Krisenpläne genutzt werden. Grundsätzlich wünschenswert wäre, wenn andere Gesundheitsämter ebenfalls After-Action Reviews ihrer während der Corona-Zeit entwickelten Strukturen durchführen und veröffentlichen würden. Dadurch könnten strukturelle Stärken und Verbesserungsbedarfe vergleichbarer gemacht werden und eventuell auch gemeinsam angegangen werden. Unser AAR zeigte, dass Ansätze, die in der akuten Notsituation als kreative Lösung dienten, wie beispielsweise die Einbindung des Personals städtischer Tochterfirmen, die Erfahrung im persönlichen Kontakt mit Menschen haben und beispielsweise aus dem Veranstaltungsmanagement über Expertise in der Lenkung großer Menschenströmen (nutzbar beim Aufbau von Impf- oder Testzentren) haben, einen Benefit zeigten und in Zukunft schneller in Aktivierungsprozesse mit eingebunden werden könnten. Wir konnten im Rahmen unserer Evaluation für viele Bereiche bestätigen, dass beschleunigte Aktivierungsprozesse das Krisenmanagement unterstützen und dies an vielen Beispielen demonstrieren: Dies kann durch das Vorhalten geeigneter, technisch gut ausgestatteter Gebäude geschehen, die im Bedarfsfall freigezogen und dem neuen Zweck zugeführt werden. Regelmäßige ämterübergreifende Abfragen zu freiwilliger Bereitschaft können helfen, für die Möglichkeit von Kriseneinsätzen zu sensibilisieren und so die Bereitschaft zum Einsatz zu verbessern. Die digitale Ausstattung ist eines der wesentlichen Elemente der Einsatzfähigkeit, wie in unserem AAR deutlich herausgearbeitet wurde.

## Limitationen

Eine Limitation des AARs ist die Kürze der Zeit, die zur Durchführung bereitstand. Ein Workshop über einen längeren Zeitraum hätte möglicherweise weitere Details hervorgebracht, deren Überarbeitung ebenfalls sinnvoll ist. Auch die Notwendigkeit zur Durchführung von Interviews in den Fällen, in denen die Teilnahme in Präsenz nicht möglich war, kann die Ergebnisse beeinflusst haben.

Insgesamt könnte eine Verschränkung des überwiegend qualitativen Formates des AAR mit quantitativen Forschungsansätzen im Sinne eines mixed-method Ansatzes zu weiteren Synergien im Erkenntnisgewinn führen.

## Schlussfolgerungen

Wir konnten zeigen, dass ein AAR von Krisenstrukturen auf kommunaler Ebene mit geringen Ressourcen durchführbar, akzeptiert und informativ ist. Die Methodik des After-Action Review eignete sich zur Erarbeitung von in einer Krisensituation aufgetretenen Herausforderungen und Lösungsansätze auf kommunaler Ebene. Die Beschreibung von Beispielen guter Praxis kann verdeutlichen, wo die Stärken der Kriseneinheit liegen um diese für notwendige Veränderungen zu nutzen. Es ist wahrscheinlich, dass im Fall zukünftiger Pandemien auf die während der COVID-19-Pandemie 2020–2023 entstandenen Strukturen zurückgegriffen wird. Die mit der Methodik des AAR erarbeiteten Herausforderungen und Lösungsansätze konnten postpandemische Handlungsfelder für die generische Pandemieplanung und das Krisenmanagement aufzeigen. Auf diese Weise kann das System im Einklang mit dem Krisenmanagementzyklus lernen und besser auf zukünftige Krisen vorbereitet sein 11 12 13 .
